# The Mediating Role of Depression in the Association Between Hearing Impairment and Functional Disability Among Middle-Aged and Older Adults in China

**DOI:** 10.1093/geroni/igad093

**Published:** 2023-08-29

**Authors:** Dan Han, Sangsang Li, Yunyi Wu, Jie Zhao, Mei Zhang, Hui Liao, Ying Ma, Chaoyang Yan, Jing Wang

**Affiliations:** Department of Health Management, School of Medicine and Health Management, Tongji Medical College, Huazhong University of Science and Technology, Wuhan, Hubei, China; Department of Health Management, School of Medicine and Health Management, Tongji Medical College, Huazhong University of Science and Technology, Wuhan, Hubei, China; Department of Health Management, School of Medicine and Health Management, Tongji Medical College, Huazhong University of Science and Technology, Wuhan, Hubei, China; Department of Health Management, School of Medicine and Health Management, Tongji Medical College, Huazhong University of Science and Technology, Wuhan, Hubei, China; Department of Health Management, School of Medicine and Health Management, Tongji Medical College, Huazhong University of Science and Technology, Wuhan, Hubei, China; Department of Health Management, School of Medicine and Health Management, Tongji Medical College, Huazhong University of Science and Technology, Wuhan, Hubei, China; Department of Health Management, School of Medicine and Health Management, Tongji Medical College, Huazhong University of Science and Technology, Wuhan, Hubei, China; Department of Health Management, School of Medicine and Health Management, Tongji Medical College, Huazhong University of Science and Technology, Wuhan, Hubei, China; Department of Health Management, School of Medicine and Health Management, Tongji Medical College, Huazhong University of Science and Technology, Wuhan, Hubei, China; The Key Research Institute of Humanities and Social Science of Hubei Province and Institute for Poverty Reduction and Development, Huazhong University of Science and Technology, Wuhan, Hubei, China

**Keywords:** Depression, Functional disability, Hearing impairment, Mediation analysis, Older adults

## Abstract

**Background and Objectives:**

This study aims to explore the association between hearing impairment (HI) and functional disability and to examine whether depression mediates this association.

**Research Design and Methods:**

In the study, 11 335 individuals aged 45 years and older were analyzed from the China Health and Retirement Longitudinal Study. The study used logistic regression and Karlson/Holm/Breen’s method to examine the correlation between HI, depression, and functional disability. Functional disability was assessed using activities of daily living and instrumental activities of daily living.

**Results:**

HI was significantly associated with activities of daily living disability (odds ratio [OR] = 1.34, 95% confidence interval [CI] = 1.21–1.49) and instrumental activities of daily living disability (OR = 1.57, 95% CI = 1.46–1.68). The mediated effect of depression accounted for 22.80% and 15.17% of the total effect of HI on activities of daily living and instrumental activities of daily living disability, respectively. Additionally, depression partially mediated the effects of HI on specific activities of daily living and instrumental activities of daily living tasks, including bathing (33.23%), toileting (27.50%), doing chores (37.36%), preparing meals (28.04%), shopping (25.81%), taking care of finances (11.82%), and taking medicine (12.71%).

**Discussion and Implications:**

HI increased the likelihood of functional disability partially through depression in middle-aged and older adults, suggesting that emphasizing the mental wellness of these people with HI is necessary to prevent impairments in physical function.

**Translational Significance:** Although hearing impairment is associated with depression and functional disability, few studies explore depression as a mediator between hearing impairment and functional disability. This study finds depression partially mediates the association between self-reported hearing impairment and functional disability, affecting different functional abilities of senior adults through depression. Interventions targeting self-reported hearing impairment and depression are crucial in preventing functional disability, particularly regarding instrumental activities of daily living among older adults.

Disability in older adults has received considerable attention and interest over the last few decades (1). The report on disability from World Health Organization (WHO) showed that 46.1% of older adults in the world (over 60 years of age) live with disability and the number is rising in parallel with the accelerated world aging process. As one of the countries with the largest aging population in the world, China has more than 42 million older adults (2). Some meta-analyses have shown that the pooled prevalence rate of disability in China is over 20% (3). WHO predicted that the number of older adults in China with disabilities will increase to 66 million by 2050 (4). According to the International Classification of Functioning, Disability, and Health (ICF), disability is the sum of impairments, activity limitations, or participation restrictions (5). As a key quality indicator for activity limitations, functional disability commonly refers to difficulty in basic activities of daily living (ADL) and instrumental ADL (IADL) (6). ADL are commonly regarded as fundamental activities for independent living at home, such as bathing, dressing, and eating (7). IADL means more complex activities that require a higher level of autonomy and cognitive function, such as taking care of finances, shopping, and preparing meals (7).

Previous studies have identified many influencing factors related to functional disability, such as sensory impairment (8,9) and mental health status (10,11). Among these factors, hearing impairment (HI) has been suggested as an important factor affecting functional disability in older adults (12). A meta-analysis study demonstrated that older adults who experienced HI were more likely to report ADL difficulties than older adults without HI (12). Some longitudinal studies showed that self-reported HI may negatively affect older adults’ physical functioning independent of baseline health status (13,14). Moreover, several studies found that older adults with HI had more limitations to specific activities than those without HI, such as walking, getting in and out of bed, and managing medication (15). However, some studies did not find an independent association between HI and functional disability (16,17).

Depression, as one of the leading causes of disability in world, was suggested as a risk factor that predicts a functional decline and physical disability in older adults (18). Kalyani et al. found that participants reporting depression showed higher functional disability in mobility tasks, doing usual activities, and heavy work (19). A cohort of community-dwelling individuals over 8 years indicated that depression was associated with increased odds of disability onset after adjusting for confounders (18). The long-term depression may reduce one’s social engagement (20), personal interaction (21), and cognitive function (22,23). Such effects are likely to limit the independent implementation of ADL and IADL (24).

Depression also has been associated with HI in related theoretical or empirical studies (25–28). The gradual development of HI is significantly linked to the reduction of personal communication capability and socialization motivation and may be more prone to emotional disorder, thus, developing into depression (27,28). Some longitudinal studies reported that the risk of developing depression is substantial in nondepressed participants with self-reported hearing problems (26,29,30). However, other studies showed that objectively measured hearing loss, but not self-reported HI, was associated with depression (31).

To date, hearing impairment is known to be independently associated with depression and functional disability, but the relationship between self-reported HI, depression, and functional disability remains inconclusive. Moreover, the studies have not further demonstrated whether depression mediates the association between self-reported HI and functional disability. Therefore, this study aims to: (i) explore the association between HI and functional disability and (ii) examine whether depression has a mediating effect in the association between HI and functional disability.

## Method

### Data Source

This study used the data of China Health and Retirement Longitudinal Study (CHARLS) in 2011, 2013, 2015, and 2018. This national survey collected information on the health and aging status of adults aged 45 years and above in China, covering a variety of information regarding basic demographic characteristics, family structure, health status, health care, etc. (32). The baseline national wave of CHARLS is being fielded in 2011 and includes 17 708 individuals in 150 counties/districts and 450 villages and committees in 28 provinces (33). Follow-up surveys were conducted every 2–3 years. As of now, CHARLS has completed regular surveys in 2011, 2013, 2015, and 2018.

The data used in this study were panel data extracted from 4 waves of CHARLS. After excluding samples with missing key variables, including self-reported hearing, depression, ADL, IADL, and confounding variables. This study finally included 11 335 participants who were followed up for all 4 waves. See Supplementary Figure 1 for details.

### Variable Measures

#### Hearing impairment

HI was accessed by the following question: “How is your hearing?” Five options were given: (1) excellent, (2) very good, (3) good, (4) fair, and (5) poor. In accordance with previous study (30), HI was categorized into 2 categories: nonimpairment (“excellent, very good, and good”), HI (“fair, poor”).

#### Functional disability

Functional disability was assessed by ADL and IADL. Five items were measured for ADL, namely, dressing, eating, toileting, bathing, and getting out of the bed. Five items were measured for IADL, namely, preparing meals, shopping, doing chores, managing assets, and taking medicine. Each answer was divided into 4 responses as follows: (i) No, I do not have any difficulty; (ii) I have difficulty but can still do it; (iii) Yes, I have difficulty and need help; and (iv) I cannot do it. Consistent with Qian and Ren 2016 (34), disability in each item was defined as participants who reported having difficultly, needing help, or cannot (ie, having a response of 2, 3, or 4). Participants were entered into an ADL or IADL disability group if they reported one of the 5 items of ADL or IADL.

#### Depression

Depression was measured by the Centre for Epidemiology Studies Depression Scale of 10 items (CES-D10). Respondents reported the frequency of occurrence of each item in the past week, and the frequency of each item was set to 0–3. The total score corresponding to the 10 items was 0–30. The higher the score was, the more severe the depression. According to recent studies, 10 as a cutoff point indicated good validity in identifying clinically significant depression (35,36). In this study, Participants were entered into a depression group if their CES-D10 score was more than 10.

#### Covariates

Covariates included sociodemographic characteristics and health-related variables. Gender (male, female), age (45–59, 60–75, 75+), marital status (married or cohabitating, single), education (illiterate, primary and middle school, high school, and above), location (urban, rural), and retirement (yes, no) and time (wave) were included in demographic characteristics. Smoking (yes, no), alcohol consumption (yes, no), chronic diseases (no, one disease, two, and more diseases), participation in social activities (yes, no), visual impairment (yes, no), the use of hearing aid (yes, no), and cognitive status (score) were considered health-related variables.

### Statistical Analysis

**Figure 1.**
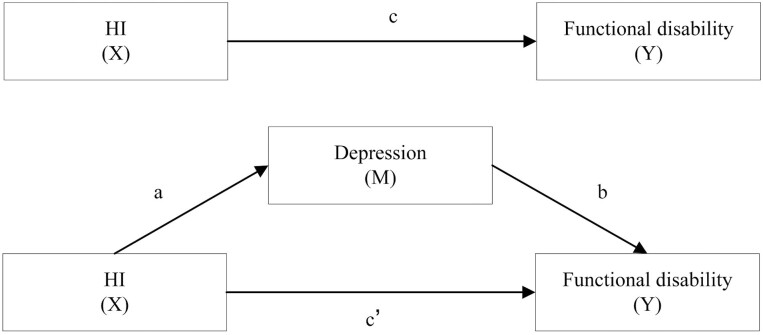
Mediating analysis of hearing impairment, depression, and functional disability. ADL = activities of daily living; HI = hearing impairment; IADL = instrumental activities of daily living. X indicates the independent variable (HI), Y indicates the dependent variable (ADL and IADL), and M indicates the mediator (depression). Mediating effect is considered if the condition is fulfilled as follows. First, X is associated with Y (path c). Second, X is associated with the M (path a). Third, M is associated with the Y after adjusting for X (path b). Finally, if the effect of X on Y is still significant when M was included in the model (path cʹ), partial mediation is considered if X is no longer significant, it is complete mediation.

First, the baseline characteristics of the participants according to hearing status were reported as percentages for categorical variables, and differences between groups were examined with the Chi-square test for categorical variables and Student’s *t*-test for continuous variables. Second, Baron and Kenny’s method for mediation analysis used the relationships between HI (X), depression (M), and functional disability (Y) (see Figure 1). Given the dichotomous nature of the functional disability in ADL and IADL and panel data of this study, binary logistic regression commands for panel data in Stata were used to examine the relationships of X, M, and Y, and the time variables were controlled. Third, the Karlson, Holm, and Breen (KHB) method is a general decomposition method that can effectively solve the scale problem related to the inseparability of estimation coefficients, through Monte Carlo simulation, the confounding percentage bias of the KHB method is smaller than other methods. The KHB logit model command for panel data was used to test the direct effect of HI (β_DE_), indirect effects (β_IE_) through depression, total effect (β_total_), and proportion of mediation (β_IE_/β_total_).

All models were adjusted for covariates listed in the variable measurement section. All data were performed using STATA 16.0, and the cutoff value of statistical significance was *p* < .05 (2-sided).

## Results

The participants’ baseline characteristics of demographic and health status are shown in Supplementary Table 1. A total of 11 335 respondents aged 45 years and above were included in this study. At baseline, 54.9% reported HI, 13.9% of participants reported ADL disability, 19.4% reported IADL disability, and 38.1% reported depression. Univariate analysis revealed significant differences prevalence of ADL disability and IADL disability in HI and depression. For covariates, hearing aid and retirement were not significant different within the ADL disability (*p* > .05) and IADL disability (*p >* .05).

### The Pathway of Hearing Impairment, Depression, and Functional Disability

**Figure 2.**
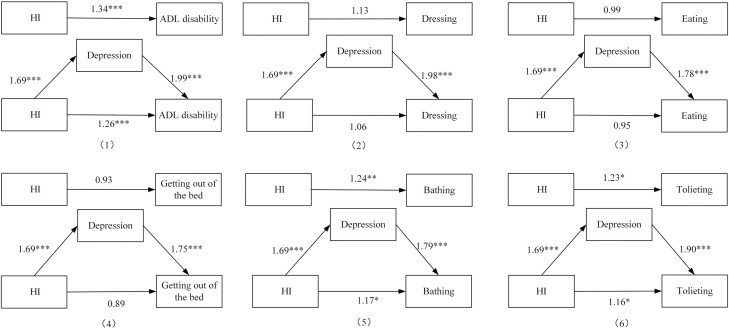
Logistic regression results of paths a, b, c, and cʹ for hearing impairment, depression, and activities of daily living disability. *p < .05, **p < .01, ***p < .001; HI: hearing impairment; ADL: activities of daily living.

Figure 2 shows the path diagram of HI and ADL disability and specific activities based on the logistic regression. For path c, HI was significantly associated with ADL disability, bathing, and toileting (*p* < .001), but not significantly associated with getting out of bed, eating, and dressing (*p* > .05). For path a, it was found a significant association between self-reported HI with depression (*p* < .001). After controlling all covariates and HI, path b showed that the depression was independently related to ADL disability, getting out of the bed, bathing, eating, dressing, and toileting (*p* < .001). Finally, when controlled covariates and depression, HI was associated with ADL disability, bathing, and toileting (*p* < .001). According to Baron and Kenny’s method, depression may have a partial mediating effect on the associations among HI and ADL disability, bathing, and toileting.

**Figure 3.**
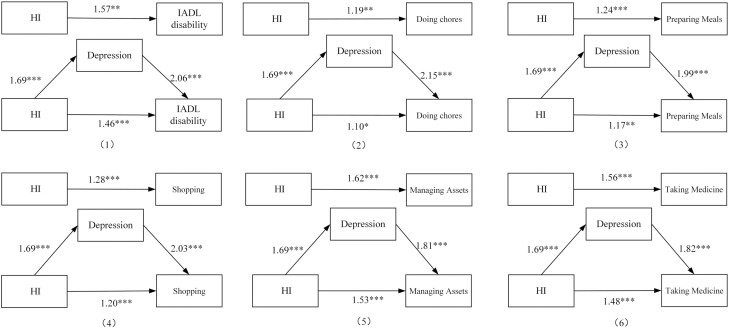
Logistic regression results of paths a, b, c, and cʹ for hearing impairment, depression, and instrumental activities of daily living disability. *p < .05, **p < .01, ***p < .001; IADL = instrumental activities of daily living; HI = hearing impairment.

Figure 3 shows the path diagram of HI and IADL disability and specific activities based on the logistic regression. For path c, HI was significantly associated with IADL disability, doing chores, preparing meals, shopping, managing assets, and taking medicine (*p* < .001). For path a, it was found a significant association between self-reported HI with depression (*p* < .001). After controlling all the covariates and HI, path b showed that the depression was independently related to IADL disability, doing chores, preparing meals, shopping, managing assets, and taking medicine (*p* < .001). Finally, when covariates and depression were controlled, HI was still associated with IADL disability, doing chores, preparing meals, shopping, managing assets, and taking medicine (*p* < .001). According to Baron and Kenny’s method, depression may have a partial mediating effect on the associations among HI and IADL disability, doing chores, preparing meals, shopping, managing assets, and taking medicine.

### Mediation of Depression of Hearing Impairment and Functional Disability

KHB mediation analysis further tested the mediating effect of depression. Tables 1 and 2 present the results of the direct effect, relative indirect effects through depression, total effect on functional disability and specific activities, and proportion of mediation. Table 1 shows that depression played a significant and partially mediating role in the relationship between HI and ADL disability, HI and bathing, and HI and toileting, respectively. Depression explained 22.80%, 33.23%, and 27.50% in the association of HI and ADL disability, bathing, and toileting, respectively.

**Table 1.** Mediation Analyses of the Association Between Hearing Impairment and Activities of Daily Living Disability by Depression

Variable	ADL Disability		Dressing		Eating		Getting Out of Bed		Bathing		Toileting	
	OR (95% CI)	*p* Value	OR (95% CI)	*p* Value	OR (95% CI)	*p* Value	OR (95% CI)	*p* Value	OR (95% CI)	*p* Value	OR (95% CI)	*p* Value
Total effect	1.34 (1.22,1.50)	<.001	1.14 (0.96,1.36)	.136	1.00 (0.76,1.32)	.973	0.93 (0.76,1.15)	.511	1.22 (1.08,1.39)	.001	1.24 (1.08,1.42)	.003
Direct effect	1.26 (1.14,1.40)	<.001	1.07 (0.90,1.28)	.432	0.96 (0.73,126)	.763	0.89 (0.72,1.09)	.252	1.14 (1.01,1.29)	.034	1.17 (1.01,1.35)	.032
Indirect effect	1.07 (1.06,1.08)	<.001	1.06 (1.04,1.08)	<.001	1.05 (1.02,1.07)	<.001	1.05 (1.03,1.07)	<.001	1.09 (1.05,1.08)	<.001	1.06 (1.05,1.07)	<.001
Mediated (%)	22.80		NA		NA		NA		33.23		27.50	

*Notes*: ADL = activities of daily living; CI = confidence interval; NA = not applicable; OR = odds ratio.

**Table 2.** Mediation Analyses of the Association Between Hearing Impairment and Instrumental Activities of Daily Living Disability by Depression

Variable	IADL Disability		Doing Chores		Preparing Meals		Shopping		Managing Finance		Taking Medicine	
	OR (95% CI)	*p* Value	OR (95% CI)	*p* Value	OR (95% CI)	*p* Value	OR (95% CI)	*p* Value	OR (95% CI)	*p* Value	OR (95% CI)	*p Value*
Total effect	1.57 (1.47,1.68)	<0.001	1.20 (1.09,1.33)	<.001	1.25 (1.14,1.39)	<.001	1.28 (1.16,1.43)	<.001	1.62 (1.49,1.77)	<.001	1.57 (1.36,1.80)	<.001
Direct effect	1.47 (1.38,1.57)	<0.001	1.12 (1.01,1.24)	.021	1.18 (1.07,1.31)	.001	1.20 (1.09,1.34)	<.001	1.53 (1.41,1.67)	<.001	1.47 (1.28,1.70)	<.001
Indirect effect	1.07 (1.06,1.08)	<0.001	1.07 (1.06,1.08)	<.001	1.07 (1.05,1.07)	<.001	1.07 (1.06,1.08)	<.001	1.06 (1.05,1.07)	<.001	1.05 (1.05,1.01)	<.001
Mediated (%)	15.17		37.36		28.04		25.81		11.82		12.71	

*Notes*: CI = confidence interval; IADL = instrumental activities of daily living; OR = odds ratio.

Table 2 shows a partially mediated effect of depression found in IADL disability and all specific activities, namely, doing chores, preparing meals, shopping, managing assets, and taking medicine, and depression explained 15.17%, 37.36%, 28.04%, 25.81%, 11.82%, and 12.71% in the association of HI and IADL disability, doing chores, preparing meals, shopping, managing assets, and taking medicine, respectively.

## Discussion

To sum up, the findings outlined a high prevalence of self-reported HI (54.9% in baseline) and depression (over 35% in baseline) in middle- and old-aged Chinese. This is consistent with the figures calculated by some recent studies on middle-aged and older adults in China (37–39), and is much higher than the average level of low- and middle-income countries (40) and even the pool prevalence of older adults globally (41). This not only warns of the daunting challenges facing China in terms of the increasing burden of HI and depression, but also reminds us of the urgent need for more accessible and feasible methods in the context of a shortage of professional resources (36,42). This finding also showed that special attention should be paid to the hearing and mental health of middle-aged and older adults in China.

This study also found an independent association between self-reported HI and functional disability, and depression had a partially mediating effect on this association. Moreover, it was found that participants with HI reported restrictions in routine activities in ADL and IADL, such as bathing, toileting, doing chores, preparing meals, shopping, managing assets, and taking medicine. A partially mediating effect of depression has also been found in these associations. These results suggested that self-reported HI may affect functional disability through depression. It is speculated that the possible reasons for these results may be related to psychology, behavior, and cognition.

### Hearing Impairment and Functional Disability

In this study, middle-aged and older adults with self-reported HI reported more difficulty performing ADL and IADL. Specifically, there was a significant correlation between self-reported HI and bathing and going to the toilet, although getting up, eating, and dressing was not associated with self-reported HI. Previous studies observed that older adults with hearing loss based on pure tone audiometry have more restrictions on getting in and out of bed than those without hearing loss (15), which is not found in this study. This may be due to different ways of hearing measurement. However, consistent with previous studies, we found that more complex IADL tasks, such as shopping, asset management, and drug therapy, were observed to be associated with HI compared with basic ADL (43). These findings suggest that self-reported HI may affect the different functional abilities of older adults and may limit their ability to perform complex functions.

According to previous studies, the decline of hearing may impair the maintenance of postural balance and change in gait and walking speed (44); thus, compared with simple self-care tasks, such as eating and dressing, older adults with HI may be restricted in movement activities that required more auditory cues (45). Further, degenerated hearing may affect cognitive and communication function. Brink and Stones found that HI was associated with impaired linguistic communication and lower levels of mood (46). As communication is an important aspect of daily living, especially when performing IADL tasks such as shopping and managing assets (47), it is not surprising that HI with its resulting communication problems would be associated with difficulty in performing these activities.

### Mediation of Depression of Hearing Impairment and Functional Disability

A notable finding in this study was that depression was reflected as a mediator in the association between HI and functional disability. Our results concur with the finding of Ye et al. that self-reported HI and depression were significantly related to functional disability (48). Further, we found that depression as a mediator can partially explain the association between self-reported HI and functional disability. This finding implied that depression may be a potential mechanism underlying the relationship between HI and functional disability. According to previous studies, there are several possible reasons for the mediated effect of depression in the association between HI and functional disability (27,49).

First, the impaired hearing was correlated with psychological disorders and health status (50). Older adults with HI are prone to negative psychology and emotion because of communication obstacles, such as anxiety and loneliness (27), and even despair when they encounter difficulties in daily life, which has been linked with the crucial risk factors for depression. According to stress theory, mental health tends to be poor when a person is stimulated by stress (49). Meanwhile, older adults with HI may experience greater health burden and higher multimorbidity rates (36), and may even receive care from family members, which leads to an increased psychological burden and produces feelings of inferiority (51).

Second, older adults with self-reported HI have more negative life events and behavior than those without HI. Older adults with HI have limited participation in social activities due to the lack of necessary hearing information to communicate with others (20). Older adults with HI may cut down on personal interaction and be predisposed to reduce the amount of time spent outside their homes and participate in social activities (52). As a result, older adults who focus on individual social roles may see themselves as a burden, which may lead to feelings of depression, and further affect physical function disability.

In addition, cognitive impairment accompanied by HI-related depression could be a possible reason for the mediation effect of depression. Some scholars have suggested that HI-related depression may affect one’s cognitive function (53), such as attention, learning domains, and visuospatial ability (54), which may cause functional disability in older adults. A recent study indicated that when older adults suffer from depression and cognitive impairment (55), their ability to perform complex aspects of ADL and specific instrumental activity may be impaired (14). Our results also indicated that depression may impair functional performance in older adults with HI, usually in more complex activities related to cognition, such as managing assets and taking medicine. Therefore, HI may affect functional disability through depression.

The current study has several strengths including a large sample size from a prospective cohort and a high participation rate, consequently giving us a great potential to draw a reasonable conclusion. However, this study still has several limitations. First, based on the CHARLS, this study only used self-reported measures for hearing, depression, and function status, rather than objective measures. However, previous evidence also found that subjective hearing indicators and depression reports can better reflect individuals’ subjective perception of their psychological and physical condition (13). Second, in view of the high prevalence and low attention of HI, this study only focused on the participants’ hearing impairment and did not explore the association of visual impairment or other sensory impairment and functional disability. However, visual impairment has been enrolled in the analysis as a control variable to avoid potential confounding. Future studies can further link different sensory impairments to functional disability and examine the mediated effect of depression. Third, this study used balanced panel data and was not a time-lag analysis, so the results cannot explain the causality. However, balancing panel data has many advantages, such as controlling the impact of omitted variables and generating more accurate predictions. Despite these limitations, the results of this study could help us better understand the relationship between HI, functional disability, and depression.

## Conclusion

Both HI and depression are associated with functional disability among middle-aged and older adults in China, and depression partially mediated the association between HI and functional disability. Moreover, HI and depression were found to be significantly correlated with specific complex tasks of ADL and IADL, including bathing, toileting, doing chores, preparing meals, shopping, managing assets, and taking medicine. Therefore, the government should take more responsibility for the early prevention of hearing impairment in middle-aged and older adults. Meanwhile, medical institutions should strengthen mental health construction when implementing intervention measures of HI, such as psychological counseling, social participation encouragement, and cognitive improvement, which may indicate changes in functional health.

## Supplementary Material

igad093_suppl_Supplementary_Material

## Data Availability

Publicly available data sets were analyzed in this study. This data can be found at: https://charls.charlsdata.com/pages/data/111/zh-cn.html and the names of the repository/repositories and accession number(s) also can be found at this link.
