# Supplementary material for: The Mediating Role of Depression in the Association Between Hearing Impairment and Functional Disability Among Middle-Aged and Older Adults in China
Source: Innov Aging. 2023 Aug 29;7(8):igad093. doi: 10.1093/geroni/igad093 (PMC10573731; doi:10.1093/geroni/igad093)
Supplement: igad093_suppl_Supplementary_Material [file igad093_suppl_supplementary_material.docx]

# Online Supplementary Material


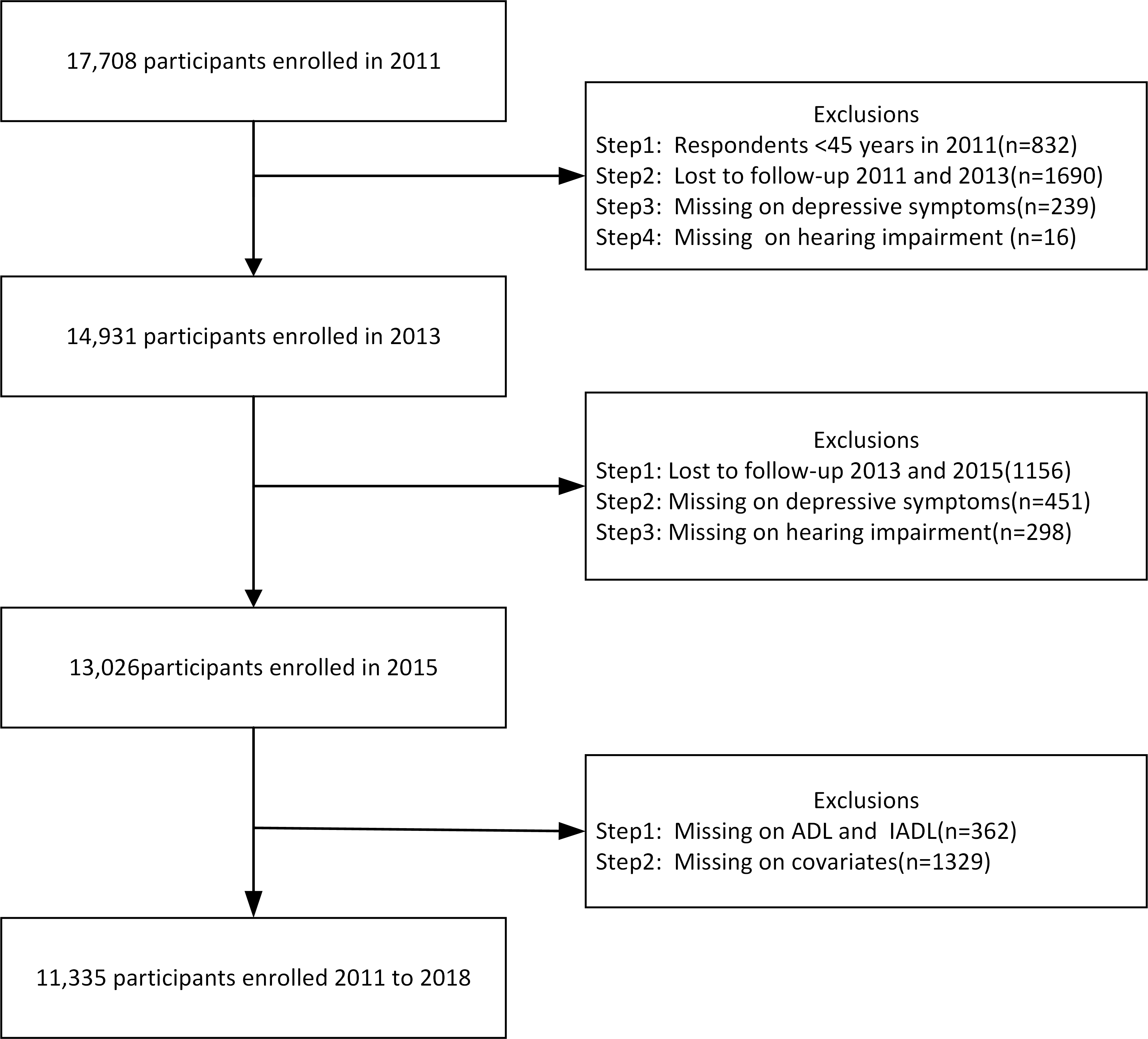


**e-figure1 Flowchart of sample selection.**

Note: ADL, activities of daily living; IADL, instrumental activities of daily living.

**e-table 1 Distribution of baseline (2011) characteristics of the participants by functional disability**

| characteristics | Total  (n=11335) | ADL Disability | | *p-value* | IADL Disability | | *p-value* |
| --- | --- | --- | --- | --- | --- | --- | --- |
|  |  | No | Yes |  | No | Yes |  |
| Sex |  |  |  | <0.001 |  |  | <0.001 |
| Male | 5,350(47.2) | 4,759(48.8) | 591 (37.3) |  | 4,536(49.7) | 814(37.0) |  |
| Female | 5,985(52.8) | 4,991(51.9) | 994(62.7) |  | 4,600(50.4) | 1,385(63.0) |  |
| Age(years) |  |  |  | <0.001 |  |  | <0.001 |
| 45-59 | 4,076(36.0) | 3,750(38.5) | 326(20.6) |  | 3,581(39.2) | 495(22.5) |  |
| 60-75 | 4,619(40,.8) | 3,985(40.9) | 634 (40.0) |  | 3,704(40.6) | 912(41.5) |  |
| >75 | 2,640(23.2) | 2,015(20.6) | 625(39.4) |  | 1,848(20.2) | 792 (36.0) |  |
| Marital status |  |  |  | <0.001 |  |  | <0.001 |
| Married | 9,477(83.6) | 8,227(84.4) | 1,250(78.9) |  | 7,730(84.6) | 1,747(79.5) |  |
| Unmarried | 1,858(16.4) | 1,523(15.6) | 335(21.1) |  | 1,406(15.4) | 454 (20.5) |  |
| Education |  |  |  | <0.001 |  |  | <0.001 |
| Uneducated | 3,071 (27.1) | 2,414 (24.8) | 657(41.5) |  | 2,123 (23.2) | 948 (43.1) |  |
| Primary and Middle school | 7,009 (61.8) | 6,153 (63.1) | 856(54.0) |  | 5,849 (64.0) | 1,160(52.8) |  |
| High school and above | 1,255 (11.1) | 1,183(12.1) | 72(4.5) |  | 1,164(12.7) | 91(4.1) |  |
| Place of residence |  |  |  | <0.001 |  |  | <0.001 |
| Urban | 2,200(19.4) | 2,016 (20.7) | 184 (11.6) |  | 1,937(21.2) | 263(12.0) |  |
| Rural | 9,135(80.6) | 7,734(79.3) | 1,401(88.4) |  | 7,199 (78.8) | 1,936(88.0) |  |
| Retirement |  |  |  | 0.063 |  |  | 0.157 |
| No | 9,876 (87.1) | 8,518(87.4) | 1,358 (85.7) |  | 7,980(87.4) | 1,896(86.2) |  |
| Yes | 1,459(12.9) | 1,232(12.6) | 227 (14.3) |  | 1,156(12.6) | 303(13.8) |  |
| Smoke |  |  |  | <0.001 |  |  | <0.001 |
| No | 6,898 (60.9) | 5,841 (59.9) | 1,057 (66.7) |  | 5,451(59.7) | 1,447 (65.8) |  |
| Yes | 4,437(39.1) | 3,909(40.1) | 528 (33.3) |  | 3,685(40.3) | 752(34.2) |  |
| Alcohol |  |  |  | <0.001 |  |  | <0.001 |
| No | 7,544 (66.6) | 6,363(65.3) | 1,181(74.5) |  | 5,932(64.9) | 1,612(73.3) |  |
| Yes | 3,791 (33.4) | 3,387(34.7) | 404 (25.5) |  | 3,204(35.1) | 587(26.7) |  |
| Chronic Diseases |  |  |  | <0.001 |  |  | <0.001 |
| 0 | 3,778(33.3) | 3,540(36.3) | 238(15.0) |  | 3,333(36.5) | 445(20.4) |  |
| 1 type | 3,432(30.3) | 3,025 (31.0) | 407(25.7) |  | 2,860(31.3) | 572 (26.0) |  |
| ≥ 2 types | 4,125(36.4) | 3,185 (31.7) | 940 (59.3) |  | 2,943(32.2) | 1,182 (53.8) |  |
| Social activities |  |  |  | <0.001 |  |  | <0.001 |
| No | 5,949(52.5) | 4,997 (51.3) | 952 (60.1) |  | 4,618 (50.6) | 1,331 (60.5) |  |
| Yes | 5,386(47.5) | 4,753 (48.7) | 633 (39.9) |  | 4,518 (49.5) | 868 (39.5) |  |
| Visual impairment |  |  |  | <0.001 |  |  | <0.001 |
| No | 10,105(89.2) | 8,835(90.6) | 1,270 (80.1) |  | 8,336 (91.2) | 1,769 (80.5) |  |
| Yes | 1,230(10.8) | 915(9.4) | 315 (19.9) |  | 800 (8.8) | 430 (19.5) |  |
| Hearing aid |  |  |  | 0.611 |  |  | 0.139 |
| Yes | 55(0.5) | 46 (0.5) | 9 (0.6) |  | 40 (0.5) | 15 (0.7) |  |
| No | 11,274(99.5) | 9.698 (99.5) | 1,576 (99.4) |  | 9,091(99.5) | 2,183 (99.3) |  |
| Cognition (Mean±SD) | 10.2±4.86 | 10.5±4.84 | 8.38±4.55 | <0.001 | 10.7±4.8 | 8.15±4.5 | <0.001 |
| HI |  |  |  | <0.001 |  |  | <0.001 |
| No | 5,110 (45.1) | 4,671 (47.9) | 439 (27.7) |  | 4,454(48.8) | 66(29.8) |  |
| Yes | 6,225(54.9) | 5,079 (52.1) | 1,146 (72.3) |  | 4,682(51.2) | 1,543(70.2) |  |
| Depression |  |  |  | <0.001 |  |  | <0.001 |
| Yes | 4,322 (38.1) | 3,259 (33.4) | 1,098 (66.8) |  | 2,960 (32.4) | 1,362 (61.9) |  |
| No | 7,013 (61.9) | 6,491 (66.6) | 546 (33.2) |  | 6,176 (67.6) | 837 (38.1) |  |

Note: ADL: activities of daily living; IADL: instrumental activities of daily living.
